# Prevalence and Influencing Factors of Coronary Heart Disease and Stroke in Chinese Rural Adults: The Henan Rural Cohort Study

**DOI:** 10.3389/fpubh.2019.00411

**Published:** 2020-01-21

**Authors:** Yan Wang, Yuqian Li, Xiaotian Liu, Haiqing Zhang, Tanko Abdulai, Runqi Tu, Zhongyan Tian, Xinling Qian, Jingjing Jiang, Dou Qiao, Xue Liu, Xiaokang Dong, Zhicheng Luo, Chongjian Wang

**Affiliations:** ^1^Department of Epidemiology and Biostatistics, College of Public Health, Zhengzhou University, Zhengzhou, China; ^2^Department of Clinical Pharmacology, School of Pharmaceutical Science, Zhengzhou University, Zhengzhou, China

**Keywords:** coronary heart disease, stroke, prevalence, influencing factors, rural population

## Abstract

**Background:** Epidemiological studies about cardiovascular disease in rural areas of developing countries are rare. This study aimed to estimate the prevalence and influencing factors of coronary heart disease (CHD) and stroke in Chinese rural population.

**Methods:** 39,259 subjects (15,490 males) aged 18–79 years were enrolled from the Henan Rural Cohort Study. Age-standardized prevalence was calculated according to Chinese 6th Population Census. Associations between risk factors and diseases were estimated by the odds ratios and 95% confidence intervals with generalized linear mixed model.

**Results:** Among the participants, 1,734 with CHD and 2,642 with stroke were identified. Crude prevalence of CHD was 4.42%, and prevalence in male (4.01%) was significantly lower than female (4.68%). Corresponding age-standardized prevalence was 2.23% (2.05% for male and 2.37% for female). Crude prevalence of stroke was 6.73%, and in male (7.92%) was higher than female (5.95%). Age-standardized prevalence was 2.98% (3.42% for male and 2.69% for female). The results identified that old age, female, smoking, obesity, hypertension, diabetes, and dyslipidemia were positively associated with CHD. Being Female and a higher level of physical activity were negatively related to stroke, while old age, high-risk drinking, and chronic disease were positively related to stroke.

**Conclusion:** CHD and stroke were not rare in Chinese rural area. Healthy lifestyles and control of chronic disease should be improved to curb the epidemic of cardiovascular disease among rural population.

**Clinical Trial Registration:** The Henan Rural Cohort Study has been registered at Chinese Clinical Trial Register (Registration number: ChiCTR-OOC-15006699). http://www.chictr.org.cn/showproj.aspx?proj=11375.

## Introduction

Cardiovascular diseases (CVD) were the leading cause of death in 2010 worldwide (1). The global deaths due to CVD have climbed from 12.59 million in 1990 to 17.92 million in 2015 (2). Of the CVD, coronary heart disease (CHD) and stroke are the major types. A systematic review reported that the prevalence of CHD across Asia, Europe, and North America ranged from 0.99 to 56.5%, and the overall prevalence was 6.3% (3), while a global study pertaining to the epidemiology of stroke have reported the prevalence ranged from 0.18 to 1.00% across central Latin America, Asia, Europe and Oceania (2).

The CVDs, major forms of chronic disease, are now becoming increasingly more common in the developing countries (4, 5). As the largest developing country, China experienced rapid health transitions. A Chinese study in 2007–2008, involving 152 cities and 112 counties, suggested that the prevalence rates of CHD and stroke were 0.63 and 0.83% (6). Another study reported that there would be an increase of ~21.3 million cardiovascular events and 7.7 million cardiovascular deaths in China from 2010 to 2030 (7).

The number of epidemiological studies of CHD and stroke in China has been increasing during the past decades. However, most of the previous studies were conducted in urban areas, much fewer in rural areas. Due to the lower educational level, lower quality of health services, and less frequency of proven therapies used, rural population might have a higher prevalence of cardiovascular disease than urban population (8, 9). To figure out the epidemiology of CHD and stroke in rural area and what mainly affect the diseases will enable the development of reasonable strategies to combat the CVD. Therefore, the aim of this study was to estimate the prevalence of CHD and stroke in Chinese rural population with exploring the related factors.

## Methods

### Study Participants

The Henan Rural Cohort Study was a large population-based study, in which rural participants were recruited from Yuzhou, Suiping, Kaifeng, Xinxiang, and Yima counties of Henan province. The baseline survey was conducted between July 2015 and September 2017. The samples were obtained by a multistage, stratified cluster sampling method. The target population in the cohort was permanent residents aged 18- to 79-year old. Forty-one thousand eight hundred and ninety-three permanent residents from administrative units (rural village) who met the inclusion criteria were invited. The exclusion criteria were that individuals suffered a severe physical or psychological disorder which made them incapable to answer the questionnaire or report to the survey location. Thirty-nine thousand two hundred and fifty-nine participants responded and the response rate was 93.7%. More detailed information of the cohort has been published elsewhere (10). The Henan Rural Cohort study has been registered in the Chinese Clinical Trial Register (Registration number: ChiCTR-OOC-15006699) before the onset of participant enrollment. All 39,259 participants were included in the final analysis. This study was approved by the Zhengzhou University Life Science Ethics Committee (Code: [2015] MEC (S128)) and was carried out according to the 1975 Declaration of Helsinki. Informed consent was signed by all study participants.

### Data Collection

In the Henan Rural Cohort Study, well-trained staff conducted a standard questionnaire by face to face interviews, to acquire information regarding demographic characteristics (age, gender, income, education, and marital status), lifestyles (high meat diet, vegetable and fruit intake, physical activity, smoking, and drinking), the history of disease and medication, family history of disease, and others. Income was divided into three levels including <500, 500~, and ≥1,000 renminbi (RMB). Education background was grouped into three categories (elementary school or below, junior high school and high school or above). Marital status was categorized into two (married/cohabitating and unmarried/divorced/widowed). High meat diet was defined as consumption of more than 75 g of meat (livestock and poultry) per day. Adequate vegetable and fruit intake was considered as the amount of vegetable and fruit consumed by a person was more than 500 g per day. Physical activity was classified as low, moderate and high level based on the International Physical Activity Questionnaire (11). Based on the smoking index (SI, SI = lifetime smoking intensity × duration of smoking) of World Health Organization (12), smoking was grouped into never smoking, light smoking (0 < SI < 200), moderate smoking (200 ≤ SI < 400) and heavy smoking (SI ≥ 400). According to the daily intake amount of alcohol of China Public Union of Nutrition and WHO, alcohol drinking was categorized as never drinking, low risk drinking (0 < ethanol/day ≤ 15 g for female, 0 < ethanol/day ≤ 25 g for male), medium risk drinking (15 < ethanol/day ≤ 40 g for female, 25 < ethanol/day ≤ 60 g for male), high risk drinking (ethanol/day > 40 g for female, ethanol/day > 60 g for male).

Weight and height were measured twice to the nearest 0.1 kg and 0.1cm, with light clothing and shoes off, by the weight device (V. BODY HBF-371, OMRON, Japan) and a standard right-angle device, respectively. Body mass index (BMI) was computed as body weight (kg) divided by height square (m^2^). BMI was divided into four levels: low weight was defined as BMI < 18.5 kg/m^2^, normal weight was defined as 18.5 ≤ BMI < 24.0 kg/m^2^, overweight was defined as 24.0 ≤ BMI < 28.0 kg/m^2^, and obese was defined as BMI ≥ 28.0 kg/m^2^ according to the criteria recommended by Working Group on Obesity in China (13). Blood pressure was measured three times by electronic sphygmomanometer (Omron HEM-7071A, Japan) in the sitting position. There were 30-s intervals between the three measurements. Hypertension was defined as the average systolic blood pressure ≥ 140 mmHg, and/or diastolic blood pressure ≥ 90 mmHg, or the use of antihypertensive medication during the last 2 weeks. The venous blood samples were collected from subjects after at least 8 h overnight fasting and the fasting blood glucose was analyzed via glucose oxidative method (GOD-PAP) by ROCHE Cobas C501 automatic biochemical analyzer. The definition of diabetes was the fasting blood glucose ≥7.0 mmol/l or having a self-reported previous diagnosis of diabetes by a physician or self-reported use of insulin or anti-diabetic medications during the last 2 weeks. Total cholesterol, triglyceride, high-density lipoprotein cholesterol and low-density lipoprotein cholesterol were estimated by Roche Cobas C501 automatic biochemical analyzer. The definition of dyslipidemia was the serum total cholesterol ≥ 6.22 mmol/L (240 mg/dl) or triglyceride ≥ 2.26 mmol/L (200 mg/dl) or high-density lipoprotein cholesterol < 1.04 mmol/L (40 mg/dl) or low-density lipoprotein cholesterol ≥ 4.14 mmol/L (160 mg/dl) or the use of lipid-lowering drug during the last 2 weeks.

### Definitions of Outcomes

All respondents were covered by the New Rural Cooperative Medical System (NRCMS), and each participant had a unique medical insurance card number and ID, making it easy to track disease. Self-reported medical histories of CHD and stroke were obtained from face to face questionnaire. Then NRCMS medical records reviews regarding CHD and stroke were confirmed by village doctor, and further identified by the outcome committee consisting of an internist, an endocrinologist, a cardiologist, and an epidemiologist according to standardization recommended by World Health Organization criteria (14, 15).

### Statistical Analysis

Continuous variables were shown as mean ± standard deviation (SD) and categorical variables were expressed in percentage. Differences in the anthropometric and demographic characteristics between groups were compared by Student's *t*-tests and chi-squared tests. The age-standardized prevalence of CHD/stroke was computed according to the data of Population Census 2010. Considering the clustering of participants in the study, a generalized linear mixed model including administrative village as the random effect variable was used to examine the association between characteristics and CHD/stroke based on odds ratio (*OR*) and 95% confidence intervals (*CI*). The model was tested with glmer{lme4}. All *P*-values were two-tailed and the significance level was 0.05. Statistical analyses were conducted by SAS 9.1 software package (SAS Institute, USA) and R software version 3.6.1.

## Results

### Characteristics of Participants

Among the 39,259 participants (15,490 male and 23,769 female), 1,734 subjects with CHD and 2,642 subjects with stroke were found. Compared with the subjects without CHD or stroke, the subjects with CHD or stroke had the following characteristics: older, lower education level, lower income, more proportion of unmarried/divorced/widowed, less high meat diet, lower high physical activity, higher BMI, more fraction of having family history of CHD or stroke, more proportion of suffering hypertension, diabetes, or dyslipidemia (Table 1).

**Table 1:** Summary statistics of the characteristics for the participants.

**Variables**	**No-CHD (*n* = 37,525)**	**CHD (*n* = 1,734)**	***p*-value**	**No-stroke (*n* = 36,617)**	**Stroke (*n* = 2,642)**	***p*-value**
Age (years), mean (SD)	55.31 (12.25)	61.87 (8.54)	**<0.001^a^**	55.00 (12.23)	63.90 (7.72)	**<0.001^a^**
Gender, *n* (%)			**0.002^b^**			**<0.001^b^**
Male	14,869 (39.62)	621 (35.81)		14,263 (38.95)	1,227 (46.44)	
Female	22,656 (60.38)	1,113 (64.19)		22,354 (61.05)	1,415 (53.56)	
Marital status, *n* (%)			**0.013^b^**			**<0.001^b^**
Married/cohabiting	33,717 (89.85)	1,526 (88.00)		33,002 (90.13)	2,241 (84.82)	
Unmarried/divorced/widowed	3,808 (10.15)	208 (12.00)		3,615 (9.87)	401 (15.18)	
Education, *n* (%)			**<0.001^b^**			**<0.001^b^**
Primary school or below	16,575 (44.17)	997 (57.50)		15,996 (43.68)	1,576 (59.65)	
Junior high school	15,090 (40.21)	553 (31.89)		14,819 (40.47)	824 (31.12)	
Senior high school or above	5,860 (15.62)	184 (10.61)		5,802 (15.85)	242 (9.16)	
Per capita monthly income, *n* (%)			**<0.001^b^**			**<0.001^b^**
<500 RMB	13,305 (35.46)	709 (40.89)		12,792 (34.93)	1,222 (46.25)	
500~RMB	12,358 (32.93)	549 (31.66)		12,123 (33.11)	784 (29.67)	
≥1,000 RMB	11,862 (31.61)	476 (27.45)		11,702 (31.96)	636 (24.07)	
Smoking, *n* (%)			**0.002^b^**			**<0.001^b^**
Never	27,292 (72.73)	1,288 (74.28)		26,793 (73.17)	1,787 (67.64)	
Light	2,144 (5.71)	62 (3.58)		2,056 (5.61)	150 (5.68)	
Moderate	1,701 (4.53)	74 (4.27)		1,638 (4.47)	137 (5.19)	
Heavy	6,388 (17.02)	310 (17.88)		6,130 (16.74)	568 (21.50)	
Drinking, *n* (%)			**<0.001^b^**			**<0.001^b^**
Never	29,021 (77.34)	1,411 (81.37)		28,417 (77.61)	2,015 (76.27)	
Low risk	5,257 (14.01)	190 (10.95)		5,091 (13.90)	356 (13.47)	
Medium risk	1,783 (4.75)	65 (3.75)		1,731 (4.73)	117 (4.43)	
High risk	1,464 (3.90)	68 (3.92)		1,378 (3.76)	154 (5.83)	
Adequate vegetable and fruit intake, *n* (%)			**0.002^b^**			0.308^b^
No	21,919 (58.41)	949 (54.73)		21,304 (58.18)	1,564 (59.20)	
Yes	15,604 (41.59)	785 (45.27)		15,311 (41.82)	1,078 (40.80)	
High meat diet, *n* (%)			**<0.001^b^**			**<0.001^b^**
No	30,272 (80.67)	1,510 (87.08)		29,457 (80.45)	2,325 (88.00)	
Yes	7,253 (19.33)	224 (12.92)		7,160 (19.55)	317 (12.00)	
Physical activity, *n* (%)			**<0.001^b^**			**<0.001^b^**
Low	12,109 (32.27)	606 (34.95)		11,551 (31.55)	1,164 (44.06)	
Moderate	14,122 (37.63)	683 (39.39)		13,906 (37.98)	899 (34.03)	
High	11,294 (30.10)	445 (25.66)		11,160 (30.48)	579 (21.91)	
Family history of CHD/stroke, *n* (%)	2,892 (7.76)	259 (15.01)	**<0.001^b^**	2,974 (8.18)	374 (14.20)	**<0.001^b^**
BMI (kg/m^2^), mean (SD)	24.81 (3.56)	25.33 (3.74)	**<0.001^a^**	24.82 (3.58)	25.05 (3.47)	**0.001^a^**
Hypertension, *n* (%)	12,035 (32.10)	804 (46.42)	**<0.001^b^**	11,252 (30.76)	1,587 (60.14)	**<0.001^b^**
Diabetes, *n* (%)	3,434 (9.17)	274 (15.85)	**<0.001^b^**	3,242 (8.87)	466 (17.68)	**<0.001^b^**
Dyslipidemia, *n* (%)	13,898 (37.08)	846 (48.90)	**<0.001**^b^	13,360 (36.53)	1,384 (52.46)	**<0.001^b^**

a *Evaluated using Student's t-tests*.

b *Evaluated using chi-squared tests*.

*CHD, Coronary Heart Disease; RMB, Renminbi; SD, Standard Deviation; BMI, Body Mass Index*.

*Bold values represent p < 0.05*.

### The Prevalence of CHD and Stroke

Crude prevalence of CHD and stroke in total participants were 4.42% (4.21–4.62%) and 6.73% (6.48–6.98%), and the age-standardized prevalence in total were 2.23% (2.08–2.38%) and 2.98% (2.81–3.15%), respectively.

The prevalence of CHD and stroke were higher among participants who were unmarried/divorced/widowed, with family history, obese, with hypertension, diabetes, or dyslipidemia. Moreover, the prevalence of CHD and stroke displayed a decreased trend as the level of education, income, and physical activity increased, while it increased with higher age (Table 2).

**Table 2:** Summary statistics of the prevalence for coronary heart disease and stroke among characteristics.

**Variables**	**CHD**	***p*-value**	**Stroke**	***p*-value**
Gender, *n* (%)		**0.002^a^**		**<0.001^a^**
Male	4.01 (3.70–4.32)		7.92 (7.50–8.35)	
Female	4.68 (4.41–4.95)		5.95 (5.65–6.25)	
Total	4.42 (4.21–4.62)		6.73 (6.48–6.98)	
Age (years), *n* (%)		**<0.001^a^**		**<0.001^a^**
18~	0.23 (0.00–0.48)		0.08 (0.00–0.22)	
30~	0.72 (0.40–1.03)		0.25 (0.07–0.44)	
40~	1.31 (1.05–1.57)		1.44 (1.16–1.71)	
50~	4.68 (4.28–5.08)		5.02 (4.61–5.43)	
60~	6.22 (5.79–6.65)		10.83 (10.28–11.38)	
70~79	7.26 (6.52–8.00)		13.79 (12.81–14.76)	
Marital status, *n* (%)		**0.013^a^**		**<0.001^a^**
Married/cohabiting	4.33 (4.12–4.54)		6.36 (6.10–6.61)	
Unmarried/divorced/widowed	5.18 (4.49–5.87)		9.99 (9.06–10.91)	
Education, *n* (%)		**<0.001^a^**		**<0.001^a^**
Primary school or below	5.67 (5.33–6.02)		8.97 (8.55–9.39)	
Junior high school	3.54 (3.25–3.82)		5.27 (4.92–5.62)	
Senior high school or above	3.04 (2.61–3.48)		4.00 (3.51–4.50)	
Per capita monthly income, *n* (%)		**<0.001^a^**		**<0.001^a^**
<500 RMB	5.06 (4.70–5.42)		8.72 (8.25–9.19)	
500~RMB	4.25 (3.91–4.60)		6.07 (5.66–6.49)	
≥1,000 RMB	3.86 (3.52–4.20)		5.15 (4.76–5.55)	
Smoking, *n* (%)		**0.002^a^**		**<0.001^a^**
Never	4.51 (4.27–4.75)		6.25 (5.97–6.53)	
Light	2.81 (2.12–3.50)		6.80 (5.75–7.85)	
Moderate	4.17 (3.23–5.10)		7.72 (6.48–8.96)	
Heavy	4.63 (4.13–5.13)		8.48 (7.81–9.15)	
Drinking, *n* (%)		**<0.001^a^**		**<0.001^a^**
Never	4.64 (4.40–4.87)		6.62 (6.34–6.90)	
Low risk	3.49 (3.00–3.98)		6.53 (5.88–7.19)	
Medium risk	3.52 (2.68–4.36)		6.33 (5.22–7.44)	
High risk	4.44 (3.41–5.47)		10.05 (8.54–11.56)	
Adequate vegetable and fruit intake, *n* (%)		**0.002^a^**		0.308^a^
No	4.15 (3.89–4.41)		6.84 (6.51–7.17)	
Yes	4.79 (4.46–5.12)		6.58 (6.20–6.96)	
High meat diet, *n* (%)		**<0.001^a^**		**<0.001^a^**
No	4.75 (4.52–4.99)		7.32 (7.03–7.60)	
Yes	3.00 (2.61–3.38)		4.24 (3.78–4.70)	
Physical activity, *n* (%)		**<0.001^a^**		**<0.001^a^**
Low	4.77 (4.40–5.14)		9.15 (8.65–9.66)	
Moderate	4.61 (4.28–4.95)		6.07 (5.69–6.46)	
High	3.79 (3.45–4.14)		4.93 (4.54–5.32)	
Family history of CHD/stroke, *n* (%)	8.22 (7.26–9.18)	**<0.001^a^**	11.17 (10.10–12.24)	**<0.001^a^**
BMI, *n* (%)		**<0.001^a^**		**0.004^a^**
Underweight	4.94 (3.56–6.32)		7.46 (5.79–9.13)	
Normal weight	3.77 (3.47–4.06)		6.00 (5.63–6.37)	
Overweight	4.54 (4.21–4.86)		7.22 (6.81–7.63)	
Obese	5.57 (5.03–6.11)		6.93 (6.34–7.53)	
Hypertension, *n* (%)	6.26 (5.84–6.68)	**<0.001^a^**	12.36 (11.79–12.93)	**<0.001^a^**
Diabetes, *n* (%)	7.39 (6.55–8.23)	**<0.001^a^**	12.57 (11.50–13.63)	**<0.001^a^**
Dyslipidemia, *n* (%)	5.74 (5.36–6.11)	**<0.001^a^**	9.39 (8.92–9.86)	**<0.001^a^**

a *Evaluated using chi-squared tests*.

*CHD, Coronary Heart Disease; RMB, Renminbi; BMI, Body Mass Index*.

*Bold values represent p < 0.05*.

### Change Tendency of CHD and Stroke Among Different Subgroups

**Figure 1:**
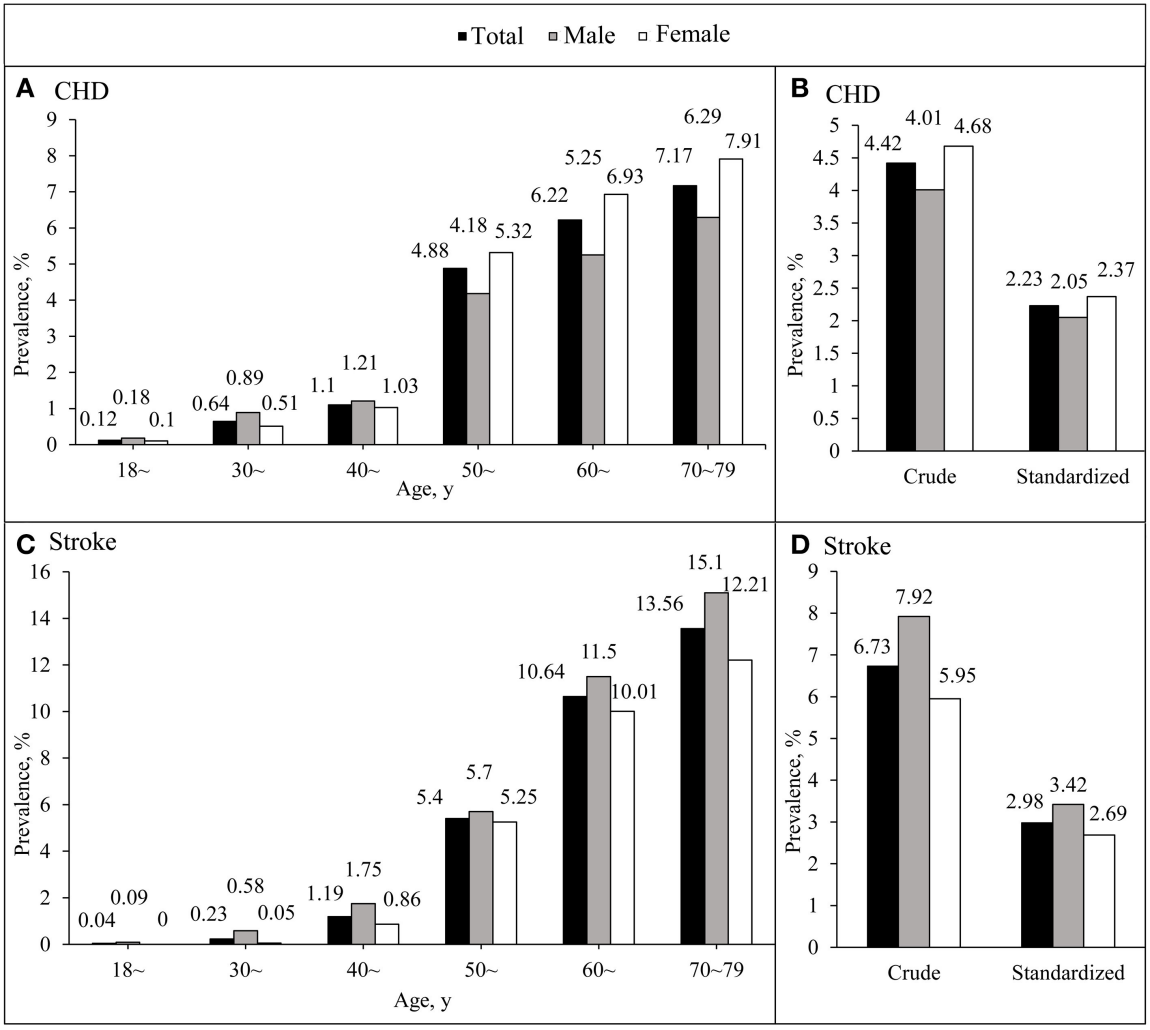
The age-standardized prevalence of coronary heart disease and stroke between different age groups among Chinese rural adults. **(A,C)** Indicate the age-standardized prevalence of coronary heart disease and stroke, respectively, according to age groups. **(B,D)** Indicate the overall crude and age-standardized prevalence of coronary heart disease and stroke.

The age-standardized prevalence of CHD and stroke increased with increasing age in both genders, with a particularly marked increase in those ≥50 years old. Among participants who were younger than 50 years old, the age-standardized prevalence of CHD in male was higher than female. Prevalence of CHD in female showed a sharp increase in the 50~ age group and exceeded that in male. In addition, the age-standardized prevalence of stroke in male was higher than female across the whole age groups (Figure 1).

Crude prevalence of CHD in male was 4.01% (3.70–4.32%), that was significantly lower than female 4.68% (4.41–4.95%). Furthermore, the crude prevalence of stroke, in male 7.92% (7.50–8.35%) was significantly higher than in female 5.95% (5.65–6.25%) (Figure 1).

### Related Factors for CHD and Stroke

Increasing age, family history, hypertension, diabetes, and dyslipidemia were all significantly positively associated with CHD and stroke. While high meat diet was significantly negatively associated with CHD and stroke. In addition, participants who were female, of smoking and obesity were more likely to suffer from CHD. High-risk drinking was positively related to stroke, while the subjects who were female and had higher physical activity were related to a lower prevalence of stroke (Table 3).

**Table 3:** The odds ratios and 95% confidence intervals of coronary heart disease and stroke.

**Variables**	**CHD**	***p*-value**	**Stroke**	***p*-value**
Age	1.06 (1.05–1.06)	**<0.001**	1.07 (1.06–1.07)	**<0.001**
Sex		**<0.001**		**<0.001**
Male	1.00		1.00	
Female	1.34 (1.13–1.59)		0.78 (0.71–0.86)	
Marital status		**0.022**		0.384
Married/cohabiting	1.00		1.00	
Unmarried/divorced/widowed	0.83 (0.71–0.97)		0.95 (0.84–1.07)	
Education				
Primary school or below	1.00		1.00	
Junior high school	0.99 (0.89–1.13)	0.996	0.98 (0.89–1.08)	0.706
Senior high school or above	1.10 (0.92–1.32)	0.278	0.93 (0.79–1.09)	0.344
Per capita monthly income				
<500 RMB	1.00		1.00	
500~RMB	1.08 (0.96–1.21)	0.223	0.92 (0.84–1.02)	0.122
≥1,000 RMB	1.02 (0.90–1.16)	0.783	0.90 (0.81–1.01)	0.055
Smoking				
Never	1.00		1.00	
Light	0.96 (0.71–1.29)	0.794	1.15 (0.93–1.42)	0.183
Moderate	1.35 (1.02–1.79)	**0.037**	1.21 (0.97–1.50)	0.091
Heavy	1.21 (0.99–1.47)	0.057	1.04 (0.89–1.20)	0.643
Drinking				
Never	1.00		1.00	
Low risk	0.94 (0.78–1.12)	0.479	0.97 (0.84–1.12)	0.675
Medium risk	0.95 (0.72–1.26)	0.733	0.98 (0.79–1.22)	0.890
High risk	1.11 (0.84–1.47)	0.472	1.38 (1.13–1.70)	**0.002**
Adequate vegetable and fruit intake	0.96 (0.86–1.08)	0.518	0.99 (0.90–1.09)	0.909
High-fat diet	0.80 (0.69–0.93)	**0.004**	0.74 (0.65–0.84)	**<0.001**
Physical activity				
Low	1.00		1.00	
Moderate	0.99 (0.88–1.12)	0.938	0.79 (0.71–0.87)	**<0.001**
High	0.83 (0.73–0.95)	**0.008**	0.61 (0.55–0.68)	**<0.001**
Family history of CHD/stroke	2.55 (2.21–2.95)	**<0.001**	2.33 (2.05–2.64)	**<0.001**
BMI				
Normal weight	1.00		1.00	
Underweight	1.20 (0.88–1.64)	0.246	1.16 (0.89–1.51)	0.274
Overweight	1.22 (1.08–1.37)	**0.001**	1.12 (1.02–1.24)	**0.019**
Obese	1.54 (1.34–1.78)	**<0.001**	1.00 (0.88–1.14)	0.998
Hypertension	1.32 (1.18–1.47)	**<0.001**	2.43 (2.22–2.66)	**<0.001**
Diabetes	1.48 (1.29–1.71)	**<0.001**	1.53 (1.36–1.71)	**<0.001**
Dyslipidemia	1.37 (1.23–1.51)	**<0.001**	1.63 (1.50–1.78)	**<0.001**
Region				
Yuzhou	1.00		1.00	
Suiping	3.83 (3.12–4.70)	**<0.001**	1.75 (1.50–2.06)	**<0.001**
Kaifeng	3.38 (2.52–4.55)	**<0.001**	1.31 (1.02–1.69)	**0.038**
Xinxiang	1.87 (1.48–2.37)	**<0.001**	0.91 (0.76–1.10)	0.324
Yima	3.59 (2.10–6.13)	**<0.001**	1.87 (1.14–3.06)	**0.013**

*All the variables in the table were adjusted in a generalized linear mixed model and age was treated as a continuous variable*.

*CHD, Coronary Heart Disease; RMB, Renminbi; BMI, Body Mass Index*.

*Bold values represent p < 0.05*.

## Discussion

The present study provided important up-to-date evidence on the current burden of CHD and stroke in a rural Chinese population. Overall, the crude prevalence of CHD and stroke in the rural population were higher than that of a previous national study in China (6). The analysis demonstrated that age, gender, high meat diet, family history of cardiovascular disease, hypertension, diabetes, and dyslipidemia were influencing factors for both CHD and stroke.

According to the finding from the 2016 global burden of disease study, age-standardized prevalence of ischemic heart disease in China was 1.51% (16). While in our study, the age-standardized prevalence of coronary heart disease was 2.23% which was higher than the results of the previous study above. In 2013, a national survey about stroke based on the National Disease Surveillance Points System, suggested that the age-standardized prevalence in total was 1.11%, in male was 1.22%, and in female was 1.01% (17). The age-standardized prevalence of stroke in the current study was 2.98% in total, 3.42% in male and 2.69% in female, demonstrating a more than 2.5-fold difference with the above study. Large variation in the prevalence of CVD exists between different socioeconomic regions and between rural and urban Chinese populations. Although, low-income regions had lower risk-factors burdens, they paradoxically had the highest CVD prevalence. And rural areas with low-income had lower risk-factors burden but similar prevalence compared with urban areas (9), which might be due to lower quality of health service and less frequent use of preventive medication (8). The higher age-standardized prevalence in the present study might suggest that the health service in rural areas were in poor condition which needs to be improved.

Old age has been reported to be associated with CVD (18). In our results, the prevalence of CHD and stroke both increased with the increasing age. And old age was positively associated with CHD and stroke in the analysis. A study has suggested that population aging would cause a dramatic increment of the incidence and deaths of CHD in China over 2010–2029 (19). Population aging might be a crucial force driving the high prevalence of rural areas. Prevalence of stroke in female was significantly lower than male and being female was negatively associated with stroke, which was in accord with a previous research showing that males were more likely to have CVD risk factors compared with females (20). However, in the present study, the prevalence of CHD among females increased dramatically when they were over 50 years old; consequently, the overall prevalence in females was significantly higher than in males. According to the results, being female was positively associated with CHD after adjusting all covariates. Menopause marks a vital cardiovascular biological transition, showing a significant increase of cardiovascular disease risk in female ≥ 50 years. A study demonstrated that the prevalence of CHD in female started to climb exponentially during the postmenopausal years (21). In the study of Women‘s Health Across the Nation (SWAN) analysis, adverse changes of lipids caused by menopausal transition were associated with the increase of CHD in postmenopausal women (22). A study showed that there are specific differences between female and male, which included that female suffer the first myocardial infarction at an older age, CHD of female always occurs after the menopause, and female are less inclined to die of CHD compared with male (23). Apart from the sex difference of biological factors, gender differences including social, educational, environmental, and community factors, also make a difference (24). Furthermore, a previous research from rural Beijing (25) showing that prevalence of stroke in males was higher than females across the age groups, while the prevalence of CHD in female aged 65 and above was significantly higher than male, which our results was in line with. In addition, the study (25) suggested that, as Chinese population ages, the burden of CHD and stroke might be different by genders, that might explain the opposite prevalence of the two diseases among males and females.

Physical activity was associated with a lower risk of CVD, this has been suggested in many investigations (26), and in our study, physical activity was related to lower prevalence of stroke and CHD indicating physical activity reduce CVD risk factors (27). Notably, we found that high meat diet was negatively associated with CHD and stroke, which were different from previous study (28). The proposition of restricting meat consumption was mainly originated from red meat as a source of saturated fatty acid, that has been considered as a risk factor of cardiovascular disease, and current dietary guidelines recommend a low-fat diet pattern and limiting saturated fatty acids intake by replacing them with unsaturated fatty acid (29, 30). However, a prospective cohort research from The Prospective Urban Rural Epidemiology (PURE) involving 18 low-income, middle-income, and high-income countries found that intake of total fat and each type of fat was related to a lower risk of total mortality, especially, higher fat intake including saturated and unsaturated fatty were associated with lower risk of stroke, and there was no observation about the harmful effect of fat intake on cardiovascular disease (31). The study (31) also demonstrated that current dietary recommendations were only based on the assumption of a linear association between saturated fatty acid intake and LDL cholesterol, and the subsequent positive relationship between LDL cholesterol and cardiovascular disease, without considering that higher intake of saturated fatty acids were also related to higher HDL cholesterol, lower triglycerides, lower total cholesterol-to HDL ratio. Besides, participants with disease might shift their unhealthy lifestyles in a beneficial direction after knowing their own physical condition, which might have influenced our results (32).

In addition, we found that high-risk drinking showed a positive association with stroke, but not CHD. Smoking, and obesity could significantly increase the odds ratio of CHD, but not stroke. In a previous study about rural Chinese population, an association between alcohol consumption and CHD or stroke was not found, while smoking could increase the odds ratio of both the CHD and stroke. Obesity was only related to CHD (33). Some differences were demonstrated. Geographical difference, discrepancy of economic level might be responsible for this phenomenon.

In the present study, hypertension, diabetes, and dyslipidemia were all positively associated with CHD and stroke that were consistent with a previous report (18). The findings indicated that the pathological status which contributed to cardiovascular disease should be timely treated and controlled.

The present study analyzed the up-to-date epidemiology of CHD and stroke in Chinese rural area. Standardized tools, well-trained professionals and the adjustment of large range of covariables helped us to control potential confounders. However, some limitations existed. Firstly, the reliable inference of causal relationship could not be drawn because of the cross-sectional design, but large researches have been conducted to demonstrate the associations between influencing factors and CHD or stroke. Secondly, the estimation of CHD and stroke prevalence mainly relied on self-report. However, the collected data were checked against NRCMS medical records reviews and were confirmed by the village doctor. What is more, the diseases were further identified by qualified internist, endocrinologist, cardiologist and epidemiologist through medical records, which guarantee the reliability of the analysis.

In conclusion, the evidence in the study demonstrated a high prevalence of CHD and stroke in Henan rural area. The treatment of hypertension, diabetes, and dyslipidemia need to be strengthened. Healthy lifestyles should be advocated for mitigating the development of cardiovascular disease and reducing disease burden in Chinese rural population.

## Data Availability Statement

The raw data supporting the conclusions of this article will be made available by the authors, without undue reservation, to any qualified researcher.

## Ethics Statement

The studies involving human participants were reviewed and approved by the Zhengzhou University Life Science Ethics Committee (Code: [2015] MEC (S128)) Zhengzhou University. The patients/participants provided their written informed consent to participate in this study.

## Author Contributions

During the research, YW had full access to all the data in the study. YW and YL analyzed the data and wrote the manuscript. CW designed the study. YW, YL, XiL, HZ, TA, RT, ZT, XQ, JJ, DQ, XuL, XD, ZL, and CW conducted the collection of the data. TA corrected the manuscript. All authors read and approve this version of the article.

### Conflict of Interest

The authors declare that the research was conducted in the absence of any commercial or financial relationships that could be construed as a potential conflict of interest.
